# TSPY-like 2, Beyond the Histone Chaperone Role

**DOI:** 10.3390/biom16030378

**Published:** 2026-03-02

**Authors:** Emanuele Bonenti, Miriana Cardano, Giacomo Buscemi, Laura Zannini

**Affiliations:** 1 Istituto di Genetica Molecolare Luigi Luca Cavalli-Sforza, Consiglio Nazionale delle Ricerche (IGM-CNR), 27100 Pavia, Italy; 2 Scuola Universitaria Superiore IUSS Pavia, 27100 Pavia, Italy

**Keywords:** nucleosome, transcription, DNA damage, cancer

## Abstract

Chromatin is a dynamic cellular structure basically constituted by nucleosomes, which consist of a DNA sequence wrapped around an octameric histones core. Histone synthesis and transport, nucleosome formation and proper chromatin assembly is an ordered and stepwise process guided by histone chaperones. Several families of histone chaperones have been identified and one of them is the nucleosome assembly protein (NAP) superfamily. Members of this family have been involved not only in chromatin constitution and regulation but also in several other cellular processes, such as nucleocytoplasmic shuttling, DNA replication, transcription and cell-cycle regulation. Testis specific protein Y-like 2 (TSPYL2) is a peculiar member of the NAP superfamily of histone chaperone. This protein has been initially isolated as a nuclear antigen in patients affected by discoid lupus erythematosus and as a TGF-β target. Its ability to bind histones has been demonstrated. In addition, TSPYL2 has been reported to regulate transcription, cell-cycle progression and the DNA-damage response, independently of its role in chromatin organization. In accordance with its multiple functions, defects in TSPYL2 have been associated with different diseases, mainly cancer and neurodevelopmental abnormalities. In this review we summarize and discuss the multiple cellular functions of TSPYL2, pointing out new and unexpected aspects like a sex-related activity and their relationship with different diseases.

## 1. Introduction

Chromatin is a dynamic cellular structure with different conformations. In eukaryotes, the nucleosome represents the simplest packaging form of chromatin and is composed of a 146 bp DNA sequence wrapped around an octameric core of histones. The octamer is composed of two copies of each histone, H2A, H2B, H3 and H4, and possesses strong affinity for DNA. The DNA connecting two histone octamers is termed ‘linker DNA’ and varies in length between 10 and 80 bp [1,2].

Nucleosomes form in an ordered manner through a complex process guided by histone chaperones [1]. Indeed, since DNA is negatively charged and histones are basic, their association is very strong, but, without histone chaperones, it could occur randomly, giving rise to irreversible and useless aggregations. In this context, histone chaperones act as modulators of histone–histone and histone–DNA interactions [3].

Different families of histone chaperones exist, consisting of various proteins grouped according to their functions. One of these is the evolutionary conserved nucleosome assembly protein (NAP) superfamily. Members of this family are all characterized by the presence of a NAP domain which is composed of a sequence of approximately 180 amino acids, sufficient for histone binding [4].

In humans, the NAP superfamily is composed of multiple members which include NAP1, the NAP1-like proteins family (NAP1L1–NAP1L5), SE translocation (SET) protein, Testis-specific protein Y-encoded family (TSPY) and testis-specific protein Y-encoded-like family (TSPYL) [5].

Proteins of the NAP family are primarily known for their role in regulating chromatin structure; however, increasing evidence indicates that they are also involved in many different cellular processes, eventually independent from histones binding. Indeed, NAP family members have been reported to participate in nucleocytoplasmic shuttling, enzyme inhibition or activation, DNA replication, transcription, gene silencing, and apoptosis [4]. Furthermore, genetic and biochemical studies have revealed that some NAPs are implicated in cell-cycle regulation and the control of mitotic events [3].

**Figure 1:**
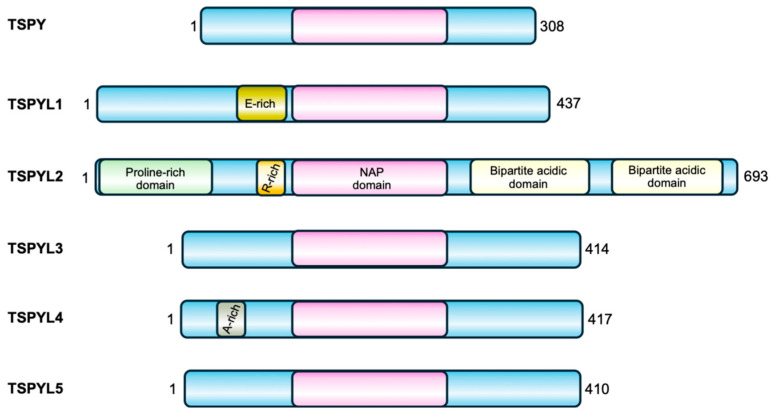
TSPY and the TSPYL family of proteins. All the members of this family are characterized by a central NAP domain (pink), surrounded by domains and sequences specific for each protein.

The *TSPYL* gene family includes six members, five coding genes (*TSPYL1*, *TSPYL2*, *TSPYL4*, *TSPYL5*, *TSPYL6*) and one pseudogene (*TSPYL3*) [5]. Except for *TSPYL2*, all other *TSPYL* genes are intron-less and located on autosomes. Their sequences and encoded proteins are not conserved apart from the NAP domain (Figure 1).

Their nucleosome assembly activity has not been fully demonstrated yet. However, these proteins have been implicated in many different biological processes including cell-cycle control, regulation of the tumor suppressor p53 function, transcriptional activation and repression, suggesting important roles in the maintenance of cell physiology. As reported in Table 1, members of the TSPYL family share common functions, such as the regulation of *CYP* genes expression (see below for details), but they may also have contrasting roles. For example, TSPY, TSPYL2 and TSPYL5 all regulate cell proliferation, but while TSPY and TSPYL5 have a positive effect on cell growth, TSPYL2 represses it. In addition, TSPYL1 negatively regulates the TGFβ pathway, while TSPYL2 promotes this signaling mechanism. Likewise, both TSPYL2 and TSPYL5 regulate p53, but while TSPYL2 is required for p53 activation, TSPYL5 was found to inhibit this protein function. Moreover, mutations or reduced expression of TSPYL members encoding genes have been associated with different diseases, as also reported in Table 1. This further confirms the specificity of their function and their involvement in different cellular processes.

Here, we review the functions and regulations of testis-specific protein Y-like-2 (TSPYL2), also known as TSPX, DENTT, CDA1 and CINAP, representing the member of the NAP family of histone chaperone whose function is best characterized. Moreover, in recent years, this protein has been gaining particular attention mainly in cancer and neurodevelopmental studies. Indeed, this protein has been demonstrated to have important roles in the regulations of gene expression, particularly of neuronal genes, and to be involved in the DNA-damage response (DDR), a complex signaling cascade that cells evolved to counteract genome instability and prevent cancer formation. Additionally, emerging roles for TSPYL2 in additional cellular events crucial for cancer prevention have recently been described.

Altogether, these findings therefore suggest that TSPYL2 constitutes an interesting topic for scientists working in different fields of biology.

## 2. TSPYL2 Discovery, Structure and Expression

TSPYL2 protein is encoded by an X-linked gene located in the short arm of X chromosome at Xp11.22 *locus* [22]. It was initially discovered as a novel nuclear antigen named Cell Division Autoantigen-1 (CDA1) during the analysis of autoimmune serum from a patient with discoid lupus erythematosus [23]. In the same year, comparing cDNA expression from transforming growth factor-β1 (TGF-β1) responsive and non-responsive epithelial non-small-cell lung cancer (NSCLC) cell lines, it was identified as a TGF-β1 target gene and therefore named Differentially Expressed Nucleolar TGF-β1 Target (DENTT) [12]. The name CASK-Interacting Nucleosome Assembly Protein (CINAP) was also attributed to TSPYL2 when it was found in complex with CASK (calcium/calmodulin-dependent serine protein kinase) and the transcription factor T-box brain 1 (Tbr-1), which are essential for cerebral cortex development [22]. TSPYL2 was also named TSPX since *TSPX* and *TSPY*, another member of the NAP chaperone superfamily, were found to have originated from homologous gene pairs on the proto-X and Y chromosomes [24].

**Figure 2:**
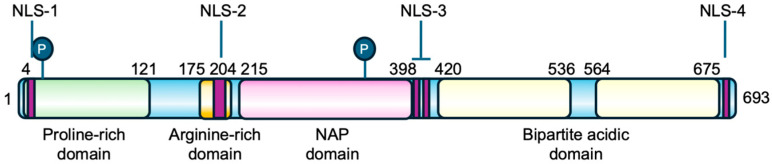
Primary structure of TSPYL2 protein. The location of TSPYL2 domains and nuclear localization signals (NLS) is indicated in the figure. In green the proline-rich domain, in dark yellow the arginine-rich domain, in pink the NAP domain, and in pale yellow the bipartite acidic domain. The circled P represents phosphorylation sites and purple boxes the nuclear localization signals.

The *TSPYL2* gene is formed by seven exons and encodes a nuclear protein of 693 amino acids structurally organized into four domains: an N-terminal proline-rich domain, an arginine-rich region and the basic NAP domain in the middle of the protein, and a C-terminal acidic domain, which also contains PEST sequences for protein stability regulation (Figure 2) [12,23]. The protein sequence is also characterized by the presence of four nuclear localization sequences (NLS): NLS-1 located at the N-terminus, NLS-2 and the bipartite NLS-3 in the central region, and NLS-4 at the C-terminal [12,23]. TSPYL2 has also been found to localize in nucleoli, a process that requires the cooperation of multiple NLSs. Specifically, NLS-2 together with either NLS-3 or NLS-4 enables the nucleolar localization of TSPYL2, indicating that NLS-2 is necessary but not sufficient for TSPYL2 targeting to these organelles. In the absence of NLS-2, the protein still enters the nucleus but is excluded from nucleoli [12].

Moreover, human TSPYL2 is estimated to be a protein of 79 kDa, but its apparent molecular weight on western blot is 130 kDa, thus suggesting the presence of extensive post-translational modifications, such as phosphorylation (Figure 2), but also acetylation or ubiquitination [12,23].

Northern blot analyses showed that *TSPYL2* is expressed in rat heart, brain, spleen, lung, liver, skeletal muscle, kidney and testis, with the highest level in the brain [22], while in mice the transcript was mainly detected in brain, testis and ovary [14]. More in detail, *TSPYL2* was found to be mostly expressed in adult mouse brain and also in regions important for neuronal progenitors production, suggesting that beside its role in the regulation of gene expression in mature neurons, TSPYL2 may also play a role in dividing cells [25].

## 3. TSPYL2 Function in Transcription Regulation

**Figure 3:**
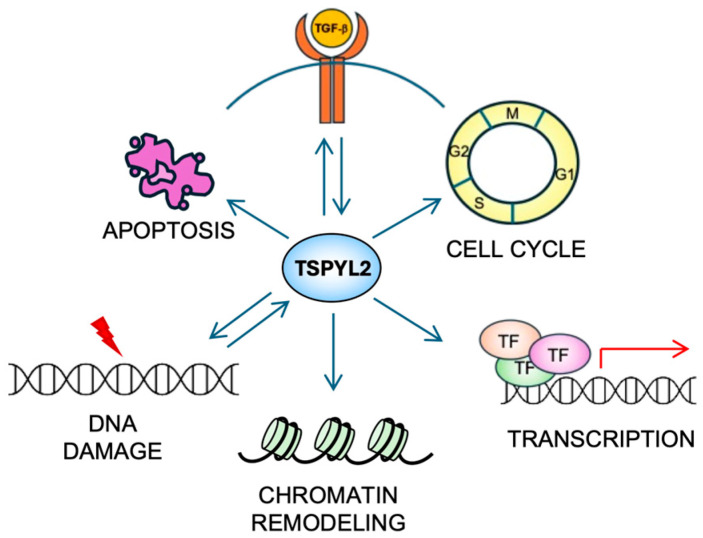
The multiple functions of TSPYL2. TSPYL2 is involved in many cellular functions (see the text for details). Indeed, it regulates cell-cycle progression, transcription, chromatin remodeling and apoptosis induction. Moreover, a reciprocal regulation (represented by back-and-forth arrows) of TSPYL2 with the TGF-β signaling and the DNA damage response has been reported.

TSPYL2 is a multifunctional nuclear protein (Figure 3) that, being part of the NAP family, influences both chromatin structure and gene expression.

In the original study, where TSPYL2 was isolated as CINAP [22], the transcriptional role of TSPYL2 was described as mediated by its interaction with histones and its nucleosome-assembly function. TSPYL2 associates with histones in regions where H4 is acetylated, suggesting its preferential targeting to transcriptionally active chromatin. Tbr-1, another CASK-interacting protein, was also found to colocalize with the TSPYL2/CASK complex in cultured hippocampal neurons, forming a tripartite protein complex which regulates the expression of Tbr-1/CASK target genes, such as NMDA receptor 2B and Reelin, finally contributing to neuronal development [22].

TSPYL2 was also reported to regulate other genes essential for neuronal differentiation and activity. For example, interacting with the transcriptional coactivator CBP, TSPYL2 directly modulates the expression of *Grin2a* and *Grin2b*, which encode the NMDA receptor subunits GluN2A and GluN2B. Accordingly, loss-of-function mutations of TSPYL2 lead to impaired long-term potentiation in hippocampal neurons and result in learning and memory deficits [16]. Moreover, TSPYL2 has been identified as a member of the RE1-silencing transcription factor/neuron-restrictive silencer factor (REST/NRSF) transcriptional repressor complex which regulates the expression of numerous neuronal genes and could also serve as tumor suppressor for various cancers [11].

Since TSPYL2 has been found in complex with histone modifiers and readers including CBP, p300, NuRD complex and the components of enhancer of zeste 2 (EZH2) complex RbAp46 and RbAp48, its role in the regulation of transcription through the modulation of histone marks has also been investigated. Intriguingly, upregulation of H3K27 trimethylation (H3K27me3), a key histone modification responsible for repressing gene expression, was found in the hippocampi of TSPYL2-KO mice, and TSPYL2 has been shown to interact with EZH2, a H3K27 methyltransferase, suggesting that it promotes expression of specific EZH2 target genes, such as *BDNF*, *EGR3* and *GRIN2C*, to support neuronal maturation and function [26].

Together with TSPYL1, TSPYL2 was also found to interact with the transcriptional co-regulator complex Z3, which consists of ZMYND8, ZNF687 and NF592, possibly contributing to the reading and interpretation of histone code for chromatin remodeling and transcription regulation [27].

The effect of TSPYL proteins on the transcription of *CYP* genes which are involved in steroid metabolism was also studied. It emerged that, while TSPYL5 regulates the expression of *CYP19A1* [18], TSPYL1, TSPYL2 and TSPYL4 bind the promoter region of *CYP2C9*, *CYP2C19*, *CYP3A4*, and *CYP17A1* thereby regulating their expression in human hepatic cell lines HepG2 and HepaRG as well as in the adrenal corticocarcinoma cell line NCI-H295R [8].

Finally, TSPYL2 was proposed to play a role in prostate cancer, as it binds and represses the androgen receptor (AR) [28], which plays important roles in testicular differentiation and spermatogenesis, as well as in the physiology and pathology of other somatic organs [6].

These findings, collectively, suggest that TSPYL2 is capable of regulating the expression of genes involved in different cellular processes, mostly in neurodevelopment, but also in metabolism and cell growth, underlining the pleiotropic functions of this protein. Moreover, these results demonstrate that TSPYL2 modulates gene expression at multiple levels. Indeed, being part of transcription complexes, it can directly influence their activity, and, through its association with histone modifiers and readers, it is also able to affect the establishment and the interpretation of the epigenome. However, the mechanisms underlying these processes still need further clarification.

## 4. TSPYL2 Interplay with TGF-β

TGF-β is a cytokine with a dual role in cancer. Indeed, in premalignant lesions it acts as a tumor suppressor by regulating cellular proliferation and apoptosis. With advancement of disease, cells learn to elude the TGF-β oncosuppressive functions, pushing this cytokine to promote tumor progression, invasion and metastasis [29].

TSPYL2 was initially identified as a TGF-β1 transcriptional target in the TGF-β-responsive NSCLC NCI-H727 cell line [12] and this finding was further confirmed in normal monkey lung bronchial cells [30] and in rodent pituitary cell line [31]. In addition, it was demonstrated that TSPYL2 suppresses the anchorage-independent growth of TGF-β responsive NSCLC cells in response to TGF-β exposure, suggesting a direct interplay between the two proteins [12]. Accordingly, overexpression of TSPYL2 was shown to increase the expression of TGF-β reporter plasmids in mouse, monkey and human cell lines [30,31,32]. Furthermore, TGF-β and TSPYL2 showed the same gene- and protein-expression pattern in human and mouse lung-tissue samples.

More recently, a study proposed that TSPYL2 exerts its tumor-suppressive function by interacting with the REST transcriptional repressor complex and promoting TGF-β signaling. In this context, REST and TSPYL2 jointly enhance TGF-β signaling by repressing the expression of genes such as the proto-oncogene *TrkC*, thereby reinforcing TGF-β-mediated growth arrest [11]. However, the role of TSPYL2 in TGF-β signaling appears to be highly context-dependent. In pancreatic adenocarcinoma it has been found that the Ski-like protein (SKIL), inhibits TSPYL2 and finally activates the TGF-β pathway, facilitating epithelial–mesenchymal transition and cellular migration [33]. Another study demonstrated that TSPYL1 and TSPYL2 play opposite roles in the regulation of TGF-β. Indeed, TSPYL1 depletion upregulates TGF-β signaling, finally increasing TSPYL2 stability which in turn interacts with SMAD complex to promote TGF-β pathway function [7]. Differently, in pulmonary fibrosis TSPYL2 plays an antifibrotic role by inhibiting the lung fibroblasts to myofibroblasts transition as well as the TGF-β pathway [34].

These reports therefore suggest that TSPYL2 and TGF-β reciprocally regulate each other (Figure 3), and that their interplay could have different impacts not only on cancer formation and progression, but also on other diseases, depending on the cellular context.

## 5. TSPYL2 and Cell Cycle Control

One of the most important roles of TSPYL2 is its involvement in cell-cycle regulation (Figure 3), particularly in G1/S and G2/M transition.

This function might be mediated by TSPYL2’s role in TGF-β regulation, but it has also been found that this protein contains two consensus sites for CDK phosphorylation (Ser20 and Thr340, Figure 2). In fact, TSPYL2 could be in vitro phosphorylated in HeLa cells by cyclin D1/CDK4, cyclin A/CDK2 and cyclin B/CDK1 [23]. In addition, TSPYL2, through its C-terminal acidic domain, also binds to cyclin B1-CDK1 complex, repressing its phosphorylative function and arresting cell-cycle progression at G2/M transition [13]. Since cyclin B1 and TSPYL2 have been found to co-localize at mitotic spindle, it has also been hypothesized that they could be required to promote an orderly G2/M transition [35]. However, the TSPYL2-dependent repression of G2/M progression occurs without affecting the integrity of the spindle-assembly checkpoint (SAC) [6].

Accordingly, overexpression of full-length TSPYL2, but not of the truncated form, arrests cell growth [23,36], and BrdU-incorporation assays showed that DNA synthesis during S-phase is repressed in TSPYL2-overexpressing cells despite the maintenance of a normal cell-cycle profile. Importantly, TSPYL2’s ability in arresting cell growth can be abolished by mutation of the two consensus sites for CDK activity [23], suggesting a fine regulation of this protein function in cell-cycle control. Indeed, it is possible that TSPYL2 phosphorylation by CDKs increases its ability to bind and inhibit cyclin B1/CDK1 complex, therefore representing a negative feedback mechanism. Of note, TSPYL2’s capacity to suppress cellular proliferation is further confirmed by its upregulation in Jurkat T cells with activated arylhydrocarbon receptor, which arrests the cell cycle [37].

It has also been found that ectopic expression of TSPYL2 inhibits cell proliferation and induces cell death in the androgen-responsive prostate cancer cell line LNCaP [38]. Conversely, another study demonstrated that TSPYL2 positively regulates cell proliferation in androgen-responsive prostate cancer cell models, demonstrating enhanced proliferation in LNCaP and 22Rv1 cell lines upon TSPYL2 overexpression [8]. Although these findings could indicate cell-specific functions for TSPYL2 in the regulation of cell proliferation, the contradictory outcomes obtained with the same LNCaP cell line highlight a significant inconsistency in the literature that requires further clarification.

## 6. TSPYL2 Involvement in the DNA Damage Response

Every day, each cell of our body is subjected to thousands of DNA lesions that, if left unrepaired, could lead to genome instability and tumor formation. To counteract the replication and propagation of damaged DNA, cells developed the DDR. The DDR is an intricate signaling network that detects DNA lesions and, depending on the damage entity, induces cell-cycle arrest and DNA repair, premature senescence or apoptotic cell death [39].

These pathways are mainly orchestrated by the ATM-CHK2 and ATR-CHK1 signaling cascades. Beyond them, many different proteins take part in these molecular mechanisms [39]. However, considering the DDR’s complexity, it is not surprising that new proteins involved in these pathways are continuously discovered.

TSPYL2 represents exactly one of the recently identified DDR players (Figure 3). Indeed, it has been demonstrated that TSPYL2 and the CDK inhibitor p21^waf1^ levels are upregulated in HeLa cells upon treatment with camptothecin, a topoisomerase inhibitor which induces DNA damage [40]. Consistently, impaired activation of G1/S checkpoint after ionizing radiation has been reported in murine embryonic fibroblasts (MEFs) derived from TSPYL2-KO mice, due to insufficient p21^waf1^ expression [14]. TSPYL2 also inactivates murine double minute 2 (MDM2), an E3-ubiquitin ligase regulating the levels of p53, a tumor-suppressor transcription factor with a central role in the DDR [40]. Indeed, in unstressed cells, MDM2 maintains p53 at low levels through ubiquitination and proteasome degradation. Following genotoxic stress, their association is disrupted and allows p53 to accumulate and become transcriptionally active. Activated p53 subsequently promotes the expression of genes required for the appropriate cellular response to DNA damage, e.g., *p21*^waf1^, *PUMA*, *NOXA* [41]. More recently, it has been found that in unstressed conditions, TSPYL2 is also maintained at low levels by MDM2-dependent protein ubiquitination and proteasome degradation, suggesting a reciprocal regulation. Upon genotoxic stress, the transcription factor E2F1 promotes *TSPYL2* gene expression and the protein, no longer associated with MDM2, accumulates and contributes to cell-cycle arrest [42]. Interestingly, this mechanism cannot be observed in male cancer cells, where the Y-encoded transcription factor sex determining region on the Y (SRY), important for male development, is frequently re-expressed. SRY expression sustains MDM2 protein levels and TSPYL2, still bound to this protein, does not escape degradation [42]. Notably, these events represent some of the earliest evidence of the involvement of sex-regulated genes and mechanisms in the DDR. Study of these processes, which play key roles in suppressing genome instability and carcinogenesis, may shed light on the sex-related disparities observed in cancer incidence, prognosis and therapeutic efficacy.

Of note, TSPYL2 was also reported to regulate p53-dependent apoptosis. Indeed, p53 function is mainly regulated through post-translational modifications (PTMs) that influence target choice and, as consequence, the cellular response to DNA damage [41]. Among PTMs an important role is played by acetylation. In particular, p53 lysine 382 acetylation, which is performed by the acetyltransferase p300 and counteracted by the NAD-dependent class III histone deacetylase SIRT1, is required for p53-dependent apoptosis induction. In the absence of DNA damage, SIRT1 maintains p300 and p53 in a hypoacetylated state to prevent programmed cell death. After genotoxic stress, TSPYL2 inhibits SIRT1 and promotes p300 activation and p53 acetylation, finally leading to apoptosis induction [15].

These results therefore suggest that TSPYL2 function is modulated in response to DNA damage, finally contributing to p53 regulation and apoptosis induction. However, considering the multiple roles of TSPYL2, we cannot exclude that, in the future, novel functions for this protein could emerge in these signaling cascades.

## 7. TSPYL2 Role in Human Diseases

Considering its essential roles in chromatin regulation, cell cycle control, and DDR, it is not surprising that TSPYL2 dysregulation contributes to the pathogenesis of diseases such as cancer and neurological disorders.

Although TSPYL2-KO mice are generally normal and do not display increased tumor predisposition [14], TSPYL2 protein is considered a tumor suppressor because of its role in preventing uncontrolled cell proliferation and maintaining genomic stability. Accordingly, TSPYL2 has mainly been found mutated in endometrial carcinoma and female-specific tumors [42,43], and downregulated in gliomas, hepatocellular carcinoma, lung, colorectal, thyroid, prostate and breast cancer. In these tumors, TSPYL2 loss is directly correlated with enhanced tumoral proliferation, migration, resistance to treatment and poor prognosis [6,44], while, on the contrary, high levels of TSPYL2 expression demonstrated oncogenic effects in colon adenocarcinoma [45].

Of note, the ten most common TSPYL2 mutations found in cancer patients are scattered throughout the protein, with hotspots falling inside the NAP and C-terminal domain (Table 2). These mutations are associated with different types of tumor, even if, in accordance with TCGA (https://www.cancer.gov/ccg/research/genome-sequencing/tcga, accessed on 20 February 2026) and [42], this protein is more commonly mutated in cancer of the uterus and in skin tumors.

Moreover, in glioma and lung cancer, TSPYL2 has been frequently found downregulated due to nitric oxide synthase-2 (NOS2)-dependent repression of *TSPYL2* gene transcription [46] as well as promoter hypermethylation [36,47]. Accordingly, treatment with demethylating agents such as 5-aza-2′-deoxycytidine restores TSPYL2 expression, highlighting an epigenetic mechanism of expression control [36]. Differently, in hepatocellular carcinoma, TSPYL2 promotes the degradation of the hepatitis B viral protein HBx via the ubiquitin–proteasome pathway, thereby counteracting the oncogenic effects of HBx and playing a tumor-suppressor role [48]. In breast and prostate cancer, TSPYL2 overexpression inhibits cellular proliferation, clonogenicity, and migration; more specifically, an induction of cell death and downregulation of oncogenic drivers such as MYC and androgen receptor (AR) was also observed in prostate cancer [38]. TSPYL2 also suppresses thyroid cancer through repression of SIRT1-AKT pathways [44] and increases gemcitabine sensitivity in pancreatic adenocarcinoma [49]. Most recently, it has also been identified as a key prognostic gene in cervical cancer, where it is found downregulated, and its overexpression inhibits malignant progression [50]. In addition, a single-cell CRISPR screening in T-cell acute lymphoblastic leukemia explored TSPYL2 role in this tumor, highlighting perturbations of STAT, NOTCH and E2F signature [51].

Beyond oncology, TSPYL2 has implications in neurodevelopmental and neuropsychiatric disorders. Indeed, TSPYL2-KO mice demonstrated impaired long-term potentiation, memory deficits, and increased levels of H3K27me3, finally linking TSPYL2 loss with dysregulation in chromatin remodeling in neurons. Furthermore, TSPYL2-KO mice display behaviors generally associated with neurodevelopmental disorders, including impaired prepulse inhibition of startle (PPI) and differences in activity and sensitivity to amphetamine, a dopamine agonist. In addition, their lateral ventricles were significantly smaller than those of wild-type (WT) mice [52]. Moreover, in humans, mutations in TSPYL2 have been linked to neurological conditions (Table 2). In fact, microduplications of Xp11.2 region, promoting copy-number variations in the *TSPYL2* gene, impact on the development of cognitive ability and speech [53]. Moreover, a study on two related boys of the Middle Eastern population of Qatar, with Autistic Spectrum Disorder, suggests a pathogenic role for their shared *TSPYL2* variant (c.1668G>C/p.Q556H) [54]. Additionally, hemizygous missense mutation in TSPYL2 (I622M) has also been found in two Pakistani boys of the same family with mild intellectual disability [55].

Because of these associations, TSPYL2 is considered a potential biomarker for early diagnosis and prognosis both in cancer and neurodevelopmental disorders, and a possible target for therapy. In addition, processes regulated by TSPYL2 may also represent potential therapeutic strategies. For example, the downregulation of neuronal EZH2 target genes observed in cells with low TSPYL2 levels can be restored through treatment with specific EZH2 inhibitors, such as GSK126 and Tazemetostat. GSK126 has already been demonstrated to increase the expression of EZH2 target genes in TSPYL2 mutant cells [26]. In contrast, Tazemetostat, an EZH2 inhibitor already approved by the FDA, has not yet been evaluated for its effects on neuronal genes expression, but it demonstrated efficacy in the treatment of follicular lymphoma in a multicenter, single arm, phase II study [56].

Besides this, TSPYL2 has recently been proposed as a biomarker for acute myocardial infarction [57] and as a possible therapeutic target for hypertension [58]. Moreover, by promoting sterol regulatory element-binding protein 2 (SREBP-2) acetylation, through SIRT1 inhibition and p300 activation, TSPYL2 plays a fibrogenic role in diabetes-associated renal injury. As such, TSPYL2 gene therapy has already been proposed for pulmonary fibrosis [34]. Finally, TSPYL2 was also reported to play a role in the pathogenesis of osteoarthritis [59]. Taken together, TSPYL2 involvement in so many different disorders could be a reflection of its multiple roles in cellular physiology.

## 8. Contrasting Properties of TSPY and TSPYL2

**Figure 4:**
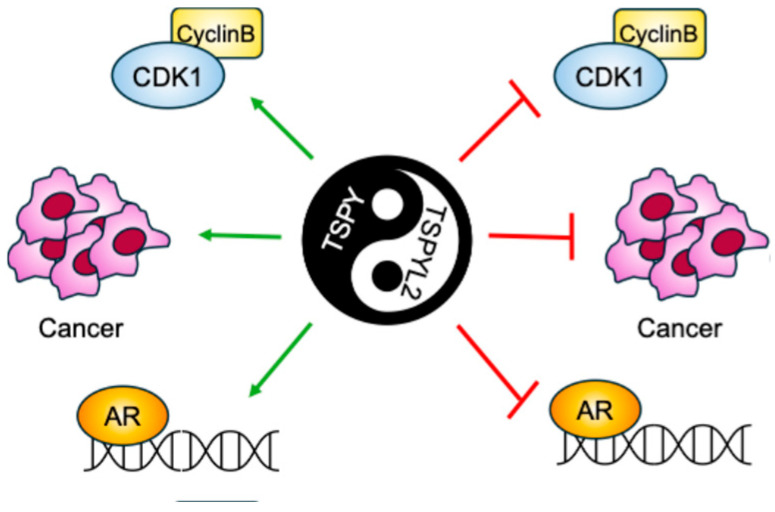
Graphical scheme of the contrasting TSPY and TSPYL2 functions. TSPYL2 was reported to inhibit Cyclin B/Cdk1 complex, tumorigenesis and androgen receptor (AR) function, while, on the contrary, TSPY was found to promote all these cellular events.

TSPY is the Y-linked homolog of TSPYL2 [6]. The gene encoding this protein is repeated 30–60 times on the male-specific region of the Y chromosome (MSY). TSPY and TSPYL2 are both characterized by the presence of the NAP domain in the same conserved exons (2–5). However, while the TSPYL2 sequence also contains exons 6 and 7 that encodes the carboxyl acidic domain, TSPY lacks such domain [6] (Figure 1). The presence of the acidic domain confers to TSPYL2 contrasting properties with TSPY (Figure 4).

Accordingly, TSPY has been described as a proto-oncogene, while TSPYL2 seems to have tumor-suppressor functions. Indeed, TSPY and TSPYL2 overexpression leads respectively to cell-proliferation promotion and arrest [13,40,60,61]. In fact, TSPY binds to type B cyclins, increasing the activity of the mitotic cyclin B-CDK1 complex and potentially inducing genome instability through premature mitosis entrance. Contrarily, TSPYL2 also associates with cyclin B, but inhibits the cyclin B-CDK1 activity. Therefore, TSPY and TSPYL2 compete for the binding to cyclin B-CDK1 and modulate the functions of this complex. The TSPYL2-mediated inhibition of cyclin B-CDK1 activity depends on its carboxyl acidic domain; accordingly, depletion of this domain allows TSPYL2 to promote cyclin B-CDK1 activity, whereas transfer of the carboxyl acidic domain to the C-terminus of TSPY represses the complex [13]. While TSPYL2 modulates cyclin B-CDK1 phosphorylation activities by maintaining the integrity of SAC, TSPY exacerbates this complex function to sustain spermatogonial stem cell renewal and male meiotic divisions. In this way, TSPY disrupts the SAC integrity ensured by TSPYL2, thereby promoting cell proliferation and oncogenesis [6].

TSPY and TSPYL2 also compete for the binding to AR [6,28]. AR co-localizes on target genes promoters with TSPY and TSPYL2, which in turn stimulate or repress their expression, respectively. These genes encode proteins implicated in cellular proliferation, cell growth, and oncogenesis, therefore emphasizing the evident different functions of TSPY and TSPYL2 respectively in promoting and suppressing the oncogenic role of AR in prostate cancer cells. As for cyclin B-CDK1 complex, the TSPYL2-inhibitory function towards AR has been located to its carboxyl acidic domain [28].

These findings therefore suggest that, although TSPY and TSPYL2 proteins differ for several domains, the disparities in their functions and their contrasting properties are all mediated by the presence of the C-terminus acidic domain in TSPYL2.

## 9. Conclusions and Future Perspectives

TSPYL2 was discovered about 25 years ago and, since then, many efforts have been made with the purpose of clarifying its role in normal cell physiology and disease. It is now well established that TSPYL2 has important functions in transcription regulation, particularly in neurons, and that it participates in DDR pathways through multiple mechanisms. In this context, its role in the regulation of the tumor-suppressor p53 is of relevance, as it may have important implications for tumor prevention and cancer therapy. Accordingly, mutations or downregulation of TSPYL2 have been demonstrated to be associated with poor prognosis in many different types of tumors, indicating for this protein a tumor-suppressor role. It is important to note that, in the future, the sex-specific regulation of TSPYL2 activity could open new possibilities for both diagnosis and personalized cancer therapy. Remarkable is also the nucleolar localization of TSPYL2, which remains unexplored, since it may be related to its tumor-suppressor function. However, it could also be interesting to address disparities in mutations frequency among different ethnicities, since, for cancer, published studies and databases are mainly focused on Western cohorts.

Moreover, given its involvement in the transcriptional regulation of neuronal genes, it is not unexpected that new evidence of TSPYL2 involvement in neurological disorders could also emerge in the next years.

We expect that new studies, aimed, for example, at the identification of TSPYL2 interacting proteins or at analyses of proteomic databases or of single cell RNA-seq results, could clarify its role in cell physiology and disease. These studies could be, however, complicated by the multifunctional nature of the TSPYL2 protein, which makes it more difficult to link defects in this protein to specific diseases. Nevertheless, insights in this context may come from studies in different model organisms and from orthologs analysis.

We are therefore confident that, in the near future, research studies will allow us to delineate the best strategies to modulate TSPYL2 activity for clinical purposes. This research will possibly lead to the development of new drugs targeting key interactions that may be relevant for novel therapeutic approaches to cancer, neurological disorders and other diseases.

## Figures and Tables

**Table 1:** Table indicating the TSPYL family members together with their reported functions.

Name	Function	Reference
TSPY	-Mainly expressed in testes -Promotes cell proliferation -Establishes a positive feedback loop with androgen receptor -Highly expressed in gonadoblastoma	[6]
TSPYL1	-Ubiquitous expression -Negatively regulates the TGFβ pathway -Regulates the expression of CYP genes -Associated with sudden infant death with dysgenesis of testes -Associated with testicular abnormalities	[7,8,9,10]
TSPYL2	-Ubiquitous expression with high levels in brain -Positively regulates and is regulated by TGFβ pathway -Restricts cell proliferation -Implicated in the DNA damage response -Promotes p53 function -Regulates the expression of CYP genes -Regulates the expression of neuronal genes -Downregulated/mutated in tumors -Associated with neurological disorders	[8,11,12,13,14,15,16]
TSPYL3	-Pseudogene -Nonfunctional	[7]
TSPYL4	-Ubiquitous expression -Regulates expression of CYP genes	[8]
TSPYL5	-Ubiquitous expression -Regulates the expression of CYP genes -Inhibits p53 function -Promotes cell proliferation and angiogenesis -Regulates alternative lengthening of telomeres -Overexpressed in tumors	[17,18,19,20]
TSPYL6	-Expressed in testes -Unknown function -Associated with breast cancer	[21]

**Table 2:** Table reporting the most common TSPYL2 mutations and the associated cancers and neurological disorders, according to COSMIC database and literature.

**TSPYL2 protein mutations**	**Cancer type**
E503K	Uterus, endometrium, lung, large intestine
S127Afs*20	Stomach, large intestine
R289C/R289S	Large intestine, lung
E590K	Skin
P33del	Large intestine
I84Sfs*63	Large intestine, stomach
S363N/S363G	Large and small intestine
R331I/R331S/R331H	Large intestine, lung, skin
A155V/A155T	Endometrium
G356R/G356E/G356W	Skin, cervix, large intestine
**TSPYL2 protein mutations**	**Associated neurological disorder**
Q556H	Autism spectrum disorder
I622M	Mild intellectual disability

## Data Availability

No new data were created or analyzed in this study.
